# European Society for Gynaecological Endoscopy (ESGE) Good Practice Recommendations on surgical techniques for Removal of Fibroids: Part 2 Hysteroscopic Myomectomy

**DOI:** 10.52054/FVVO.16.4.054

**Published:** 2024-12-17

**Authors:** T.J. Clark, L Antoun, A Di Spiezio Sardo, V Tanos, J Huirne, E.W. Bousma, T Smith-Walker, E Saridogan

**Affiliations:** Birmingham Women’s & Children’s Hospital, University of Birmingham, United Kingdom; Obstetrics and Gynaecology Department, Università degli Studi di Napoli “Federico II”, Italy; Department of Basic and Clinical Science, University of Nicosia Medical School and Aretaeio Hospital, Cyprus; Department of Obstetrics & Gynecology, St. Antonius Hospital, The Netherlands; Department of Obstetrics & Gynaecology, Amsterdam University Medical Center and Amsterdam Reproduction and Development Research Institute, The Netherlands; Gynaecology Department, Royal Cornwall Hospital, United Kingdom; Elizabeth Garrett Anderson Institute for Women’s Health, University College London and University College London Hospital, United Kingdom

## Abstract

Submucosal uterine fibroids are the rarest type of fibroids. They can lead to abnormal uterine bleeding and may play a role in infertility and miscarriage. Hysteroscopic myomectomy is the preferred treatment to relieve bleeding caused by these fibroids and to restore the normal structure of the uterine cavity. The European Society for Gynaecological Endoscopy Uterine Fibroids Working Group developed recommendations based on the best available evidence and expert opinion for the surgical treatment of uterine fibroids. In this second part of the recommendations, hysteroscopic approaches are described. This review explores the techniques related to hysteroscopic myomectomy, focusing on narrower scopes, fluid management and advances in tissue removal systems and electrosurgery.

## Introduction

Hysteroscopic myomectomy is considered the first-line treatment option in the surgical management of submucosal fibroids because it is the least invasive approach to myomectomy, avoiding surgical incisions and preserving the integrity of the outer uterine wall. Hysteroscopic myomectomy is safe and effective in treating heavy menstrual bleeding (HMB) with a success rate of between 70% to 99% (Emanuel et al., 1999; Hart et al., 1999; Vercellini et al., 1999). Failure to alleviate menstrual symptoms after hysteroscopic myomectomy is thought to arise from the ex-novo development of fibroids, the presence of fibroids sited elsewhere within the uterus, other aetiologies for HMB such as concomitant adenomyosis, and/ or where the submucosal fibroids are incompletely removed (AAGL., 2012; Van Dongen et al., 2006; Di Spiezio Sardo et al., 2008).

Several authors have assessed the impact of hysteroscopic myomectomy on reproductive outcomes in infertile women although the limitations of research designs limit clinical inferences. The data we have from one small randomised controlled trial (RCT) (Casini et al., 2006; Bosteels et al., 2015) and observational data (Pritts et al., 2009) seems consistent with a doubling of the clinical pregnancy rate with removal of fibroids but any effect on live birth or miscarriage rates remains uncertain. This lack of certainty may in part be attributed to the difficulty of controlling the many confounding factors that may account for infertility, as well as inadequate study sample sizes, limited duration and completeness of follow-up, and variation in the characteristics of the fibroids treated (i.e. number, size, intramural extension and the coexistence of multiple intramural fibroids).

Whilst we need higher quality data regarding the effectiveness of hysteroscopic removal of submucosal fibroids on abnormal uterine bleeding and fertility, the focus of this guideline is on describing the surgical techniques. Pre-operative and post-operative care and fluid management are briefly described because successful surgery cannot be achieved without attention to these aspects of care. However, this guidance concentrates on informing the reader about the surgical approaches to conducting successful hysteroscopic myomectomy. The definitions of treatment setting; office, outpatient and operating room (inpatient/ day-case/ambulatory) are described according to the International Consensus Statement for recommended terminology describing hysteroscopic procedures (Carugno et al., 2021).

## Materials and methods

In this guideline, we will refer to the 2013 revision of the FIGO classification, which to date is the most widely used system for describing the location of fibroids in clinical practice (Table I) (Wamsteker et al., 1993; Munro et al., 2011). The classification recognises that the relative degree of myometrial involvement is the primary determinant of both the feasibility and prognosis associated with hysteroscopic myomectomy. The STEP-W (“size, topography, extension, penetration, and wall”) classifications (Lasmar et al., 2005) is a more comprehensive preoperative classification of submucous fibroids where a score is generated indicating the degree of surgical complexity, but this is not widely used in clinical practice to date (Table II). Limitations of this classification include how fibroid size is weighted in the scoring system; fibroids of 2cm through to 5 cm are considered homogeneous, but in reality, the fibroid volumes will vary markedly and as a result the likely surgical complexity. Furthermore, the impact, if any, on surgical difficulty of low, middle or upper cavity fibroid location is unclear. Whilst the Lasmar classification does not specifically address the feasibility of hysteroscopic myomectomy in an outpatient or office setting (Carugno et al., 2021), it is intuitive that Group 1 (low complexity) procedures should be selected, as prolonged procedures are less likely to be achievable or tolerated by the conscious patient.

**Table I:** FIGO classifications of submucosal fibroids (modified from Munro et al., 2011). This classification was based on the original European Society for Gynaecological Endoscopy Classification (Wamsteker et al., 1993). Type 3 fibroid refers to those which are entirely intramural but contact the endometrium. See Saridogan et al. (2024) for the full classification of other type fibroids).

Type	Cavity: Myometrial relationship	Hysteroscopy
0	100% within the cavity (0% in the myometrium)	Pedunculated
1	>50% within the cavity (<50% in the myometrium)	Angle <90° between the fibroid attachment to the cavity surface
2	</= 50% within the cavity (>/ =50% in the myometrium)	Angle >90° between the fibroid attachment to the cavity surface

**Table II:** STEPW classification of uterine fibroids (modified from Lasmar et al., 2012).

	Size (cm)	Topography	Extension of the base	Penetration	Lateral wall	Total
0	< 2	Low	< 1/3	0	+1	
1	2 to 5	Middle	1/3 to 2/3	<50%		
2	>5	Upper	>2/3	>50%		

Score	Group	Complexity and therapeutic options
0 to 4	I	Less complexity hysteroscopic myomectomy
5 to 6	II	High complexity hysteroscopic myomectomy Consider GnRHa use Consider two steps hysteroscopic myomectomy
7 to 9	III	Consider alternatives to the hysteroscopic techniques

The main surgical technologies and techniques used in contemporary gynaecological practice to undertake hysteroscopic myomectomy are described below. Less commonly performed methods such as the use of Nd:yAG or diode lasers are not described.

### Fluid management

A detailed discussion on fluid management is beyond the scope of this guideline. There are two detailed guidelines covering the management of fluid distension media in hysteroscopic surgery (Umranikar et al., 2016; AAGL., 2013) which we would draw the attention of the reader to. Whilst the use of gravity and pressure cuffs can be used to instil fluid into the uterine cavity, these methods are only appropriate for diagnostic and simple, short operative procedures such as targeted biopsy and polypectomy. Successful, and importantly safe, removal of submucosal fibroids by hysteroscopic means necessitates good visualisation. This can only be achieved reliably by the use of automated fluid management systems (e.g. Aquilex®, Fluent®, Hysterobalance®, Hysterolux® and Hysteromat® systems) that provide constant irrigation to remove blood / debris and consistent intrauterine pressures. It is important to keep the intrauterine pressure below mean arterial pressure to minimise systemic absorption of fluid; the lowest irrigation pressure that allows adequate visualisation should be chosen typically starting at 75-80mmHg and increasing up to a maximum of 120mmHg.

The other main reason for using automated fluid management systems for hysteroscopic myomectomy is because the procedure can be associated with serious complications arising from excessive intravasation of fluid. This results in fluid overload within the systemic vascular circulation and electrolyte disturbances. These fluid complications are more likely when large or multiple fibroids with significant intramural involvement. Thus, meticulous peri-operative monitoring of fluid deficit is mandatory to avoid potentially life-threatening complications from systemic absorption of fluid. Automated fluid management systems are the only way of reliably measuring fluid deficit in real time. Physiological (Normal) saline is less likely to induce hyponatraemia and should be used with mechanical or bipolar electrosurgical methods. Monopolar electrosurgery necessitates the use of a non-conducting fluid medium such as sorbitol or glycine. Greater care is needed with hypo-osmolar solutions such as these. For healthy patients the maximum, acceptable fluid deficit thresholds at which the hysteroscopic myomectomy procedure should be immediately stopped is 2.5L for isotonic media such as Normal saline and 1L for non- isotonic media (Umranikar et al., 2016; AAGL., 2013).

### Pre-operative planning and preparation

#### Pre-operative planning

The aim of hysteroscopic myomectomy, regardless of the surgical modality used, is complete enucleation of the submucosal fibroid, leaving behind no residual fibroid tissue. Diagnostic work-up is important to counsel women as well as plan their hysteroscopic treatment with the aim of optimising both procedural and clinical outcomes. Radiological imaging and hysteroscopy should be considered complimentary tests, although they will provide some overlapping information.

#### Imaging

Imaging in the form of 2D and 3D pelvic ultrasound with or without contrast is important to assess the number, size (dimensions) and location of fibroids. The FIGO type of submucosal fibroid can be estimated, assessing the relative degree of intramural involvement and for FIGO type 2 fibroids, the distance from the serosal surface. The greater this “myometrial free margin” the more feasible complete fibroid removal is likely to be. Magnetic Resonance Imaging (MRI) can also be used but is more expensive and should be considered second line (Namkung et al., 2001).

#### Hysteroscopy

**Figure 1:**
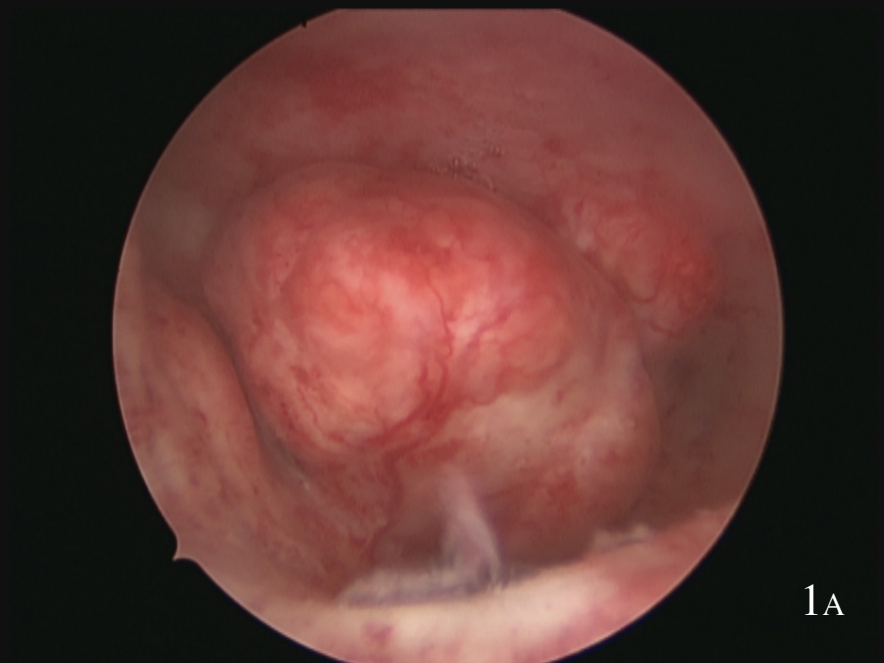

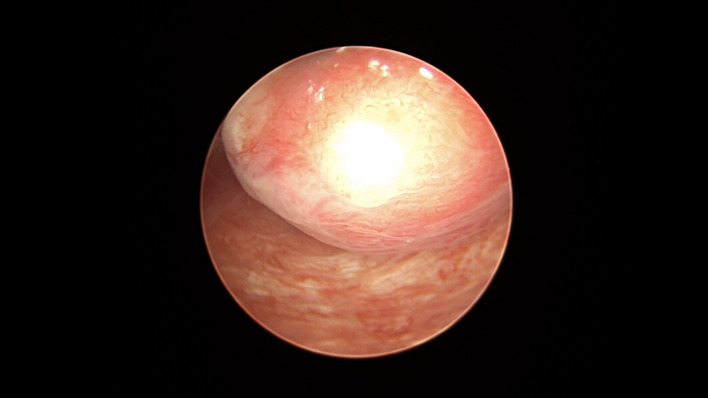

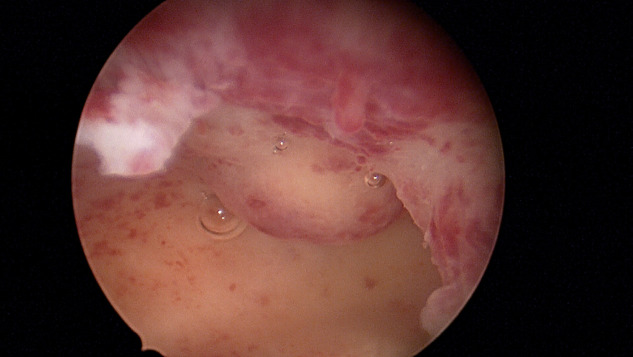
Types of submucosal fibroids. A. Type 0 fibroid, note that the fibroid is entirely intracavitary. B. Type 1 fibroid, more than 50% of the cavity is intracavitary, an acute angle of < 90ᵒ can be seen on the right side and the largest diameter of the fibroid is within the cavity. C. Type 2 fibroid, less than 50% is intracavitary, note obtuse angles of >90ᵒ can be seen on both sides.

Direct endoscopic visualisation of a submucosal fibroid is considered the gold standard diagnostic test (Farquhar et al., 2003; van Dongen et al., 2007). However, it should be noted that type 2 fibroids may be pushed into the myometrium with the pressure of distension fluid during hysteroscopy and may be overlooked. Therefore, the entire cavity should be assessed carefully when the distension fluid is turned off to see if any fibroids protrude into the cavity, after the initial assessment with fluid distension. Hysteroscopy allows key characteristics of the submucosal fibroid to be recorded such as estimates of its size, the intramural proportion of the fibroid (based upon the angle between the intracavity fibroid and endometrial surface; acute angle < 90° = type 1 and obtuse angle > 90° = type 2 fibroid (Wamsteker et al., 1993), surface area relative to the uterine sidewall and overall cavity, surface vascularity and location (Figure 1). Assessment of these features helps the surgeon plan treatment including the need for down regulation, the best technology to employ and the most appropriate treatment setting. It also allows counselling of women about the potential need for multi-step procedures. Hysteroscopy in the context of planning fibroid treatment should be performed in an outpatient / office setting where possible (De Silva et al., 2024).

### Pre-operative preparation

#### Medical preparation

The decision to employ pre-operative hormonal treatment should be left to the surgeon’s discretion, as existing data on surgical feasibility (e.g., duration, completeness), safety (e.g., fluid absorption, uterine trauma), and prognosis (clinical outcomes) remains inconsistent when comparing hormonal preparation with the absence of pre- operative medical intervention (Gulmann et al., 2005; Campo et al., 2005; Mavrelos et al., 2010; Muzli et al., 2010). Regarding endometrial or fibroid preparation, practice varies among surgeons; some never employ pre-surgical treatment, while others utilize it routinely or selectively, depending on the anticipated surgical complexity and fibroid size. Some surgeons also strategically schedule procedures to coincide with the thinnest endometrial phase during the proliferative stage of the menstrual cycle.

Where medical treatment is deemed appropriate, a 2–3-month regimen prior to surgery is recommended to achieve endometrial downregulation. Hormonal treatments available include progestogens, danazol, gonadotropin- releasing hormone analogues (GnRH-a), and gonadotropin-releasing hormone antagonists (GnRH-ant), each inducing amenorrhea. This pre- operative amenorrheic state offers the benefit of allowing patients to take iron supplements and optimize iron stores, potentially lowering surgical morbidity. Additionally, surgical visibility is improved in the absence of blood, endometrial debris, and congestion. GnRH-a treatments are particularly effective in thinning the endometrium and reducing both the volume and vascularity of submucosal fibroids by inducing a hypoestrogenic state.

Conversely, GnRH antagonists, such as relugolix, elagolix, and linzagolix, achieve adequate haemoglobin levels and reduce both myoma and uterine size by suppressing the hypothalamic-pituitary-ovarian axis and decreasing sex steroid levels without the flare-up effect (Muzii et al., 2024). These agents, with the additional benefit of oral administration, have been shown to reduce heavy menstrual bleeding and induce amenorrhea within a month (Neblett et al., 2023; Di Spiezio Sardo et al., 2023). Furthermore, an additional advantage of using GnRH antagonists is the possibility of combining them with add-back therapy (e.g., relugolix with low-dose oestrogen and progestin). This solution enables the mitigation of hypoestrogenic side effects, enhancing both tolerability and patient comfort, thereby promoting adherence to the pre-operative treatment regimen. Although data on surgical outcomes remains limited, preliminary findings indicate that GnRH antagonists offer promising alternatives to traditional treatment options.

#### Surgical preparation

Surgical preparation in the outpatient / office hysteroscopy clinic has been described and uses a 5Fr bipolar electrode to incise over the endometrial mucosa covering the fibroid, proceeding along the reflection line on the uterine wall, up to the cleavage plane between the fibroid and its pseudo- capsule (Bettocchi et al., 2009; Haimovich et al., 2013). The hope is that subsequent myometrial contractions will partially or wholly expel the intramural component of the fibroid rendering them more accessible and amenable to removal. However, efficacy data are lacking to recommend the routine use of this approach.

Vasopressin induces a vasoconstrictive effect on tissues and is used liberally in abdominal approaches to myomectomy (Kongnyuy et al., 2008; Kongnyuy et al., 2014). Its use is encouraged in the European Guidelines as well (Umranikar et al., 2016). However, studies on the use of vasopressin in hysteroscopic myomectomy are limited. Some studies have shown that the intracervical administration of vasopressin reduces the amount of blood loss and distension fluid absorbed due to its vasoconstrictive effect (Goldenberg et al., 1996; Philips et al., 1996). Some clinicians have investigated the direct hysteroscopic injection of diluted vasopressin into the submucosal fibroid using a 5Fr hysteroscopic needle (Holloran- Schwartz et al., 2014; Rouholamin et al., 2021; Philips et al., 1996). Data are limited, but a small randomised controlled trial showed that that the injection of vasopressin during hysteroscopic myomectomy reduced surgical time, fluid deficit and improved visualisation (Phillips et al., 1996; Rouholamin et al., 2021). More research regarding the effectiveness of this intervention is needed to guide practice.

#### Radiological preparation

The use of immediate pre-operative uterine artery embolisation has been described to devascularise large submucosal fibroids, typically greater than 4.5cm in maximum diameter, facilitating their removal (Namkung et al., 2018). In the absence of more data, embolisation should only be used in selective cases or within a research context.

#### Cervical preparation

There is no compelling evidence to support the routine use of cervical preparation with oestrogen (post-menopausal women), prostaglandins (e.g. misoprostol), osmotic dilators (e.g. laminaria tents) to enhance the feasibility, safety or patient experience of hysteroscopic myomectomy (AAGL, 2012; Al Fozan et al., 2015; Phillips et al., 1997).

Clinicians may decide to selectively use such measures where cervical dilatation beyond Hegar 6 (6mm) is anticipated (Cooper et al., 2011).

#### Antibiotics

Antibiotics should not be routinely prescribed prior to hysteroscopic procedures, including hysteroscopic myomectomy. Whilst endometritis following resectoscopic myomectomy has been reported to effect 1 in 200 women (Agostini et al., 2002), there is no evidence to support the routine use of antibiotics to prevent genital tract or systemic infection (Agostini et al., 2002; Bhattacharya et al., 1995).

### Fibroids without intramural involvement (FIGO type 0)

Hysteroscopic removal of fibroids without an intramural component are the least technically challenging procedures because the whole fibroid is visible within the uterine cavity and so more easily accessible. However, they can be more mobile than FIGO type 1 and 2 fibroids which can create some challenges, especially when adopting the technique of resectoscopic slicing. The risks of inadvertent uterine trauma and fluid overload are lower because operating within the deeper myometrium, where larger diameter blood vessels are present.

Several surgical techniques and technologies have been proposed for the removal of fibroids without an intra-myometrial component. These techniques are described in the three sections below.

#### Resectoscopic slicing of fibroid

The slicing technique, generally referred to as transcervical resection of fibroid (TCRF) (Hart et al., 1999; Vercellini et al., 1999; AAGL, 2012; Di Spiezio Sardo et al., 2008) is performed with a standard 22 Fr (7mm) or 26 Fr (~9mm) resectoscope or, more recently, with a 15 Fr /16fr (~5mm) resectoscope. The latter, often referred to as “mini-resectoscopes”, enable removal of smaller type 0 myomas, typically < 2.5 cm in maximum diameter, without prior cervical dilation and, in many cases, this can be performed in an outpatient / office setting (Grimbizis et al., 2021).

The “slicing” technique involves repeated passes of the cutting loop to gradually remove slices of fibroid tissue until it has all been removed. The cutting loop is placed beyond the distal border of the fibroid and repeated, systematic, retrograde electrosurgical cutting movements, facilitated by movement of the hysteroscope or withdrawal of the cutting loop or a combination of both methods, are undertaken until the fibroid is removed. Resection is usually initiated at the free margin of the myoma and proceeds in a uniform manner towards its base of implantation within the myometrium. Attempts may be made during the procedure to coagulate specific bleeding points using the loop if the bleeding persists and is compromising visualisation. Coagulation of the superficial and visible vessels can be also performed prior to resection, as a preventive action to optimise visualisation and potentially diminish fluid intravasation.

The resected slices or ‘chips’ are most commonly removed using blind polyp forceps and / or by trapping them onto the end of the hysteroscope by retracting the inactivated cutting loop and removing the resectoscope. Care should be taken during blind removal as this may potentially increase risk of uterine perforation. Repeated instrument insertions are usually required. In the case of small lesions, retrieval of the resected tissue can be done at the end of the procedure. For larger fibroids, removal of free-flowing ‘chips’ of tissue will be required on one or more occasions during the procedure to maintain an adequate view. Some surgeons avoid the formation of free flowing ‘chips’ of tissue by removing each resected strip of tissue by immediate removal of the resectoscope followed by re-insertions. Whilst this approach maintains a view, it can be laborious, and repeated loss of uterine distension and tamponade can temporarily lose view, contains a risk of air embolism (Conner and Clark, 2020; Brooks et al., 1997) and prolongs the procedure. Another option for removing resected fibroid tissue from the uterine cavity is by aspiration. An evacuator (e.g. the Urovac bladder evacuator) is attached to the outer sheath of the resectoscope after removal of the working element, and pieces of detached fibroid tissue are aspirated in a manner similar to that used by urologists when undertaking transurethral resection of prostate (TURP).

**Figure 2:**
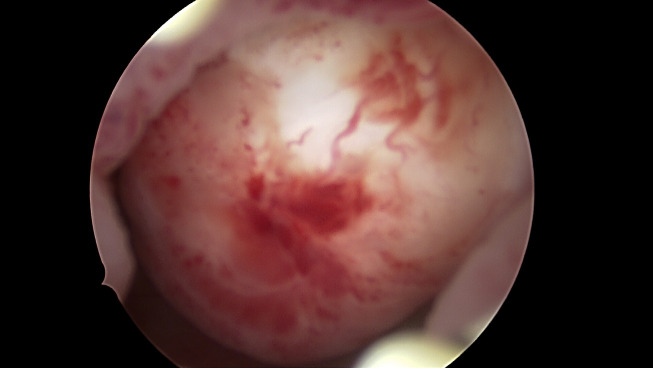

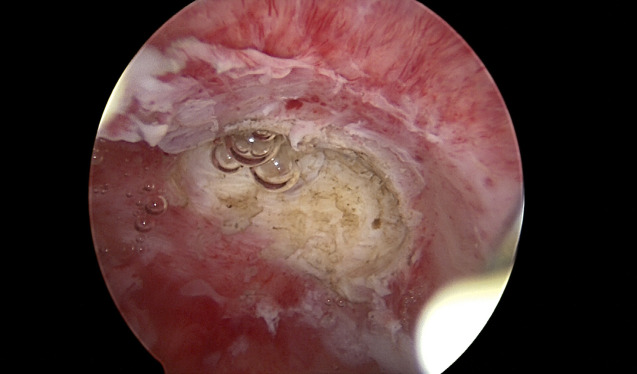
Type 0 fibroid before (A) and after (B) resection.

On completion of detachment of the fibroid and removal of all tissue retained within the cavity, the final step is to inspect the base where the fibroid was attached, to ensure complete enucleation (Figure 2). Myometrial tissue is identified as pinker, softer tissue and muscular fascicles should be visible. The inactivated cutting loop can be used to help judge the tissue density and delineate the myometrial base (fibroid capsule). The activated electrode is used to remove any residual fibroid tissue from the base. During this final step, the hysteroscopist must be particularly careful and meticulous in adjusting the depth of each cut making sure that iatrogenic thermal injury to healthy myometrium or surrounding endometrium is prevented.

Resectoscopic slicing of FIGO type 0 fibroids can be performed in both outpatient/office and operating room settings but in general smaller fibroids are amenable to outpatient/office removal with smaller diameter ~5mm mini-resectoscopes.

#### 5Fr electrode slicing of fibroid

This method utilises a straight 5Fr electrode passed down the working channel of a continuous flow operating hysteroscope of 4.5-5.5mm outer diameter. The 5Fr electrode is placed at the distal extremity of the fibroid and a retrograde surgical cut is made to the proximal extremity. Repeated cuts are made in the same furrow so that the fibroid is sliced vertically until the basal attachment with the myometrium is reached (Clark et al., 2002; Varma et al., 2009; Bettocchi et al., 2002). Depending on the shape of the fibroid (sphere or ovoid), it can be first divided into two hemispheres with a 5 Fr bipolar electrode, and then each half is cut into fragments using systematic vertical and / or horizontal slices. The cut pieces should be small enough to remove with hysteroscopic grasping forceps.

5Fr electrode slicing can be used for FIGO type 0 fibroids, typically no more than 1.5cm in maximum diameter. The technique was developed for use in an outpatient/office setting but can be adopted in the operating room setting to minimise the need for cervical dilatation associated with the use of larger diameter conventional resectoscopes and mechanical hysteroscopic tissue removal (mHTR) systems.

#### Electrosurgical en-bloc removal of fibroid

The electrode (5Fr straight, or resectoscopic cutting loop/knife) is placed at the level of attachment of the fibroid to the myometrium and beyond its distal border (Clark et al., 2002; Varma et al., 2009; Bettocchi et al., 2002). Retrograde electrosurgical cutting is then performed by movement of the hysteroscope or withdrawal of the electrode or a combination of both methods. Repeated anterograde, or preferably retrograde (to minimise the risk of thermal uterine perforation), cutting motions are continued in a systematic way until the fibroid is detached (these may proceed in one direction or may be done alternatively from each free border until they meet centrally). The inactivated electrode can be used periodically to probe the fibroid to help identify the basal attachment to the myometrium.

The electrode can be used to slice the fully or partially detached fibroid into smaller pieces to aid removal from the cavity (Bettocchi et al., 2002) using hysteroscopic or blind instruments like polyp forceps. In practice, it is difficult to morcellate a fibroid that has been partially or fully detached from the myometrium due to its mobility. Alternatively, the fibroid can be retrieved from the uterine cavity in one piece. This usually necessitates variable degrees of cervical dilatation and blind insertion of instruments such as polyp forceps. Wherever possible, blind instrumentation of the uterus should be avoided to minimise the risk of genital tract trauma. Another option for blind removal, or when blind removal is not easily achieved, is to leave the detached fibroid in situ to degenerate and pass spontaneously post- operatively and often during the first menstruation after surgery (Varma et al., 2009; Haimovich et al., 2015). An outpatient hysteroscopy 6-8 weeks later can be considered to see if there is any residual necrotic fibroid tissue, which can be removed using hysteroscopic instruments or a mHTR. Finally, a ‘hybrid’ procedure can be performed where a mHTR is used to remove under direct vision after complete or partial detachment of the fibroid (Munro, 2016).

Electrosurgical en-bloc removal of FIGO type 0 fibroids can be performed in both outpatient/office and operating room settings but in general smaller fibroids are amenable to outpatient/office removal with smaller diameter 4.5-5.5mm operative hysteroscopes accommodating 5Fr electrodes and ~5mm mini-resectoscopes.

#### Vaporisation of the fibroid

Vaporisation of the fibroid is performed using ball- shaped (spherical) or barrel-shaped (cylindrical) electrodes, which are passed in a retrograde fashion slowly along the surface of the fibroid (Brooks, 1995), inducing its complete disintegration. The depth of vaporisation depends on the duration of contact, as well as the resistance and power of the generator. It is important to move the electrode slowly along the tissue, applying current only when the electrode is being retracted toward the operator. Any pressure exerted too long on a single spot may result in uterine perforation. High power outputs are required, which produce gas bubbles that can enter the vascular system although they generally dissipate rapidly in the blood. A constant monitoring of the patient’s end-tidal CO2 together with close cooperation between the surgeon and anaesthetist is recommended to avoid serious complications from gas emboli. The advantage of this technique is the avoidance of chip formation.

However, the main drawback of using vaporising electrodes is that no tissue is left for histological examination.

Ablation by retrograde passage of an Nd:yAG laser over the surface of a submucosal fibroid </=2 cm in diameter has been described (Di Spiezio Sardo et al., 2008; Istre et al., 2009). However, like vaporisation, the duration of the procedure and the lack of a tissue specimen for pathologic evaluation are disadvantages. Vaporisation of fibroids is generally performed in an operating room setting.

#### Mechanical Hysteroscopic Tissue Removal (mHTR) of fibroid

mHTR systems provide a means of mechanical cutting of fibroid tissue with simultaneous aspiration of this tissue, allowing a clear surgical view and a specimen for histological examination. The available mHTR systems vary between 5mm and 7.25mm in outer diameter and the integral operating channels vary in diameter between 3 and 4 mm, down which the cutting blades are passed. Blades or “shavers” designed for cutting dense tissue are selected for removing fibroid tissue.

The technique requires firm contact of the instrument tip with the submucous fibroid prior to activation. The cutting window is placed within the central portion of the fibroid to create a ‘bite’ (likened to eating an apple), followed by lateral rotations of the cutting window (likened to spreading butter on toast) (Emanuel and Wamsteker, 2005; van Dongen et al., 2008). These manoeuvres are repeated until the fibroid removed. Minimal anterograde or retrograde movements of the HTRS are required and these are only to ensure that the cutting window is placed within fibroid tissue at all times to ensure efficient removal.

Mechanical hysteroscopic tissue removal of FIGO type 0 fibroids can be performed in both outpatient/office and operating room settings but in general smaller fibroids are amenable to outpatient/ office removal using mHTR systems </= 6.25mm.

### Fibroids with intramural component (FIGO Type 1 & 2)

Hysteroscopic treatment of fibroids with an intramural component is technically more challenging and associated with a higher risk of complications, especially uterine trauma and fluid overload. When planning hysteroscopic myomectomy, the size of FIGO type 1 and 2 fibroids is the most important characteristic to appreciate as regards feasibility and safety. As a general rule, FIGO type 1 and 2 fibroids that are planned to be removed hysteroscopically should not exceed 4–5 cm in maximum diameter.

Several surgical techniques have been proposed for the removal of fibroids with a myometrial component that cannot be seen. All techniques have the common objective of trying to produce an intracavitary projection of the previously intramural component, facilitating direct and safe cutting and removal. These techniques are described in the following sections “Resectoscopic slicing of fibroid, ‘Cold loop’ myomectomy, 5Fr electrode en-bloc removal of fibroid and Mechanical Hysteroscopic Tissue Removal of fibroid”. In addition, non-surgical measures can be considered to induce myometrial contractions, facilitating the migration of the ‘hidden’ intramural component of a fibroid into the uterine cavity, thus making hysteroscopic resection safer and more feasible. Myometrial contractions may be induced manually, hydrostatically and pharmacologically and are applicable to all surgical methods described in the sections below. Hydro-massage involves inducing changes in intrauterine pressure by intentionally interrupting and restarting the instillation of distension fluid repeated times. Manual massage can also be used to compress and stimulate the uterus. Reported pharmacologically aided techniques include transabdominal injection of prostaglandin F (PGF)-2a under laparoscopic monitoring and intracervical injection of carboprost, a methyl analogue of PGF-2a. These techniques are generally not practised in isolation but in conjunction with the previously described techniques (Di Spiezio Sardo et al., 2008; Istre et al., 2009).

#### Resectoscopic slicing of fibroid

Removal of the intracavity component of the fibroid by resectoscopic slicing is described in the ‘Fibroids without intramural component (FIGO type 0) section. Removal of the fibroid is continued with slicing of the portion enveloped within the muscle of the uterine wall, until it is completely removed. The technique is described below.

As soon as the visible intracavity portion of the fibroid has been removed, the inactivated cutting loop is gently used, exerting care to avoid damaging the fragile loop, to undermine the myometrial component of the fibroid in order to separate it from the underlying capsular attachment (Hart et al., 1999; Vercellini et al., 1999; AAGL., 2012; Di Spiezio Sardo et al., 2008; Istre et al., 2009). Whilst mechanical separation may not be possible with the relatively delicate loop, such manoeuvres help distinguish fibroid tissue, which appears as smooth, white and compact, from the underlying, softer and pinker myometrium. The capsule within the myometrium from where the fibroid is enucleated, is identifiable as a ‘crater’. This ‘defect’ begins to become apparent as visible bridges of filmy, slender connective tissue that ‘hold’ the fibroid in place within the myometrium are disrupted by stretching, tearing or cutting using manipulations of the inactivated and activated cutting loop. Clear delineation of the cleavage plane (the space between the fibroid and the adjacent myometrial tissue), allows the remaining fibroid tissue within the myometrium to be removed systematically, minimising the risk of inadvertent uterine perforation, using deeper, retrograde electrosurgical cuts into residual fibroid tissue. Loss of uterine tamponade by deliberately reducing intracavity distension pressure, or as occurs when retrieving chips of tissue, induces myometrial contraction and this can facilitate further expulsion of the intra-myometrial component of the fibroid into the uterine cavity.

**Figure 3:**
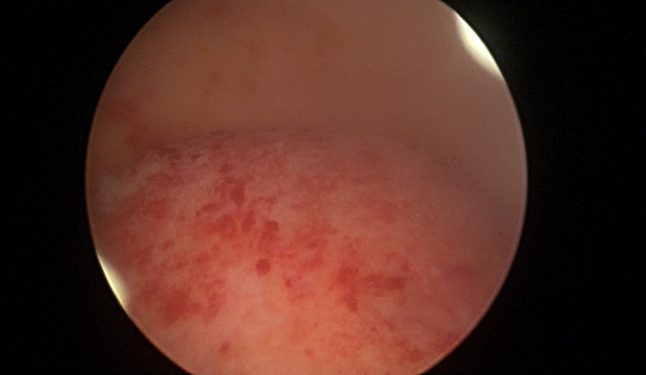

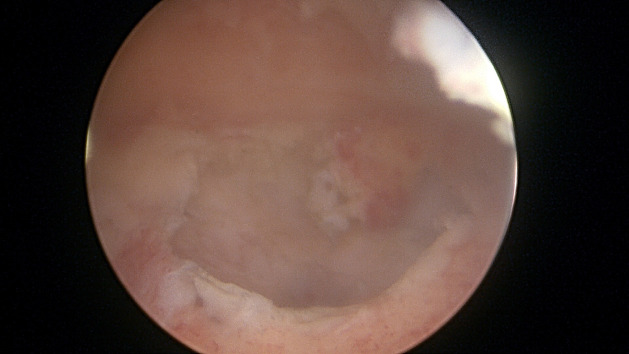
Type 2 fibroid before (A) and after (B) resection.

The careful use of the activated and inactivated electrosurgical loop is continued in this manner along the cleavage plane until the fibroid has been enucleated (Figure 3). Attempts may be made to coagulate specific bleeding points during the procedure using the loop if the bleeding persists and is compromising visualisation. However, deeper myometrial vessels are of larger diameter such that bleeding may only be limited using greater fluid distension to tamponade vessels. Meticulous fluid balance (see ‘Fluid Management’ section above) must be kept as rapid intravasation of fluid can occur the deeper into myometrial tissue the fibroid extends (Umranikar et al., 2016; AAGL, 2013). Removal is as described in the section for FIGO type 0 fibroids.

Techniques to preserve endometrium during resection of fibroids with a significant intramural component (FIGO Type II and III) fibroids have been described in women with an intention of future pregnancy to reduce the potential risk of intrauterine adhesions and thin endometrium (Di Spiezio Sardo et al., 2016). This technique comprises either making an endometrial incision or making a small opening in the overlying endometrium to expose the cleavage plane between the fibroid and myometrium. Subsequent steps of resection are then performed through this small opening without resecting the overlying endometrium (Vorona and Saridogan, 2022).

#### ‘Cold loop’ myomectomy

This technique, developed by Mazzon et al. (2015) is an adaptation of conventional electrosurgical loop resection. Electrosurgical resection continues in the described fashion until the level of the plane of the endometrial surface. The cleavage plane is then identified. Subsequent enucleation of the intramural component of the fibroid is then undertaken using more robust, bespoke ‘cold’ loops designed for mechanical blunt dissection. Hard rectangular and single-toothed loops can be used to separate, hook and lacerate the slender connective bridges of myometrial tissue which join the fibroid and the adjacent myometrium. Once the intramural part of the fibroid is totally separated within the uterine cavity, standard electrosurgical resection using an angled cutting loop completes the procedure as described in the previous section (Mazzon et al., 2015).

Resectoscopic slicing of FIGO type 1 and 2 fibroids is generally performed in an operating room setting under regional or general anaesthesia due to the longer duration of procedures and the need to manipulate within the myometrium.

#### 5Fr electrode en-bloc removal of fibroid

**Figure 4:**
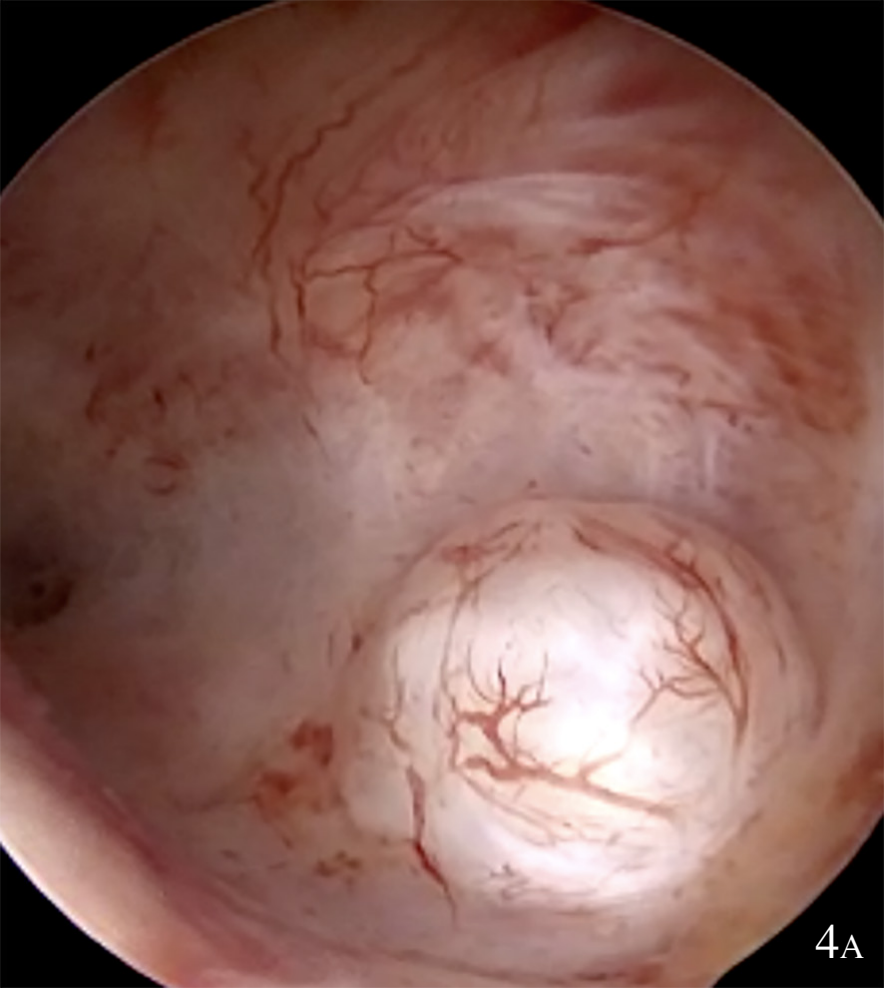

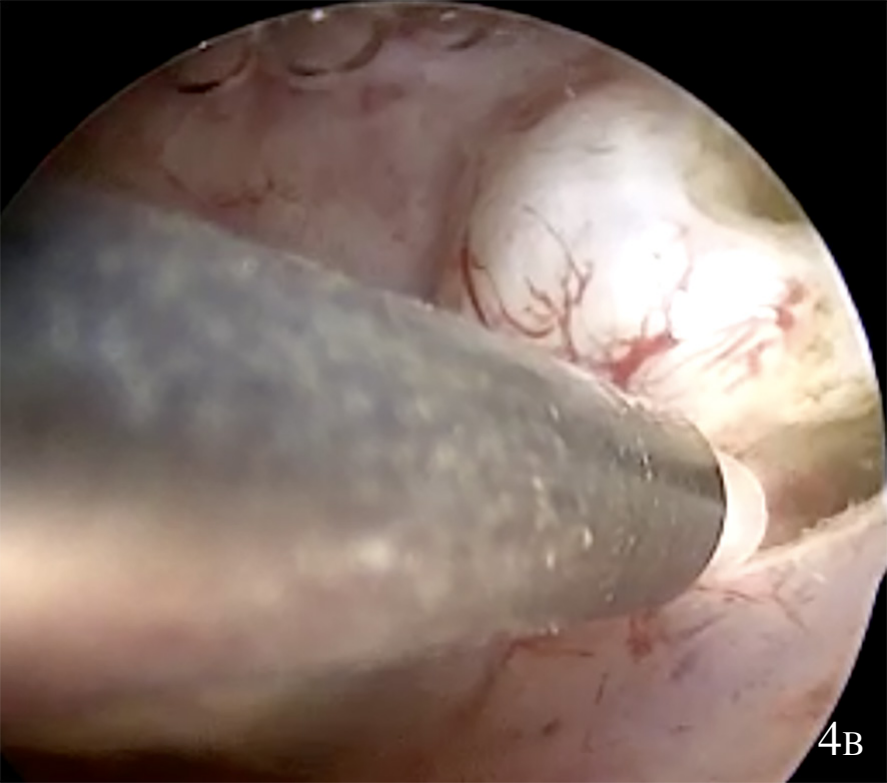

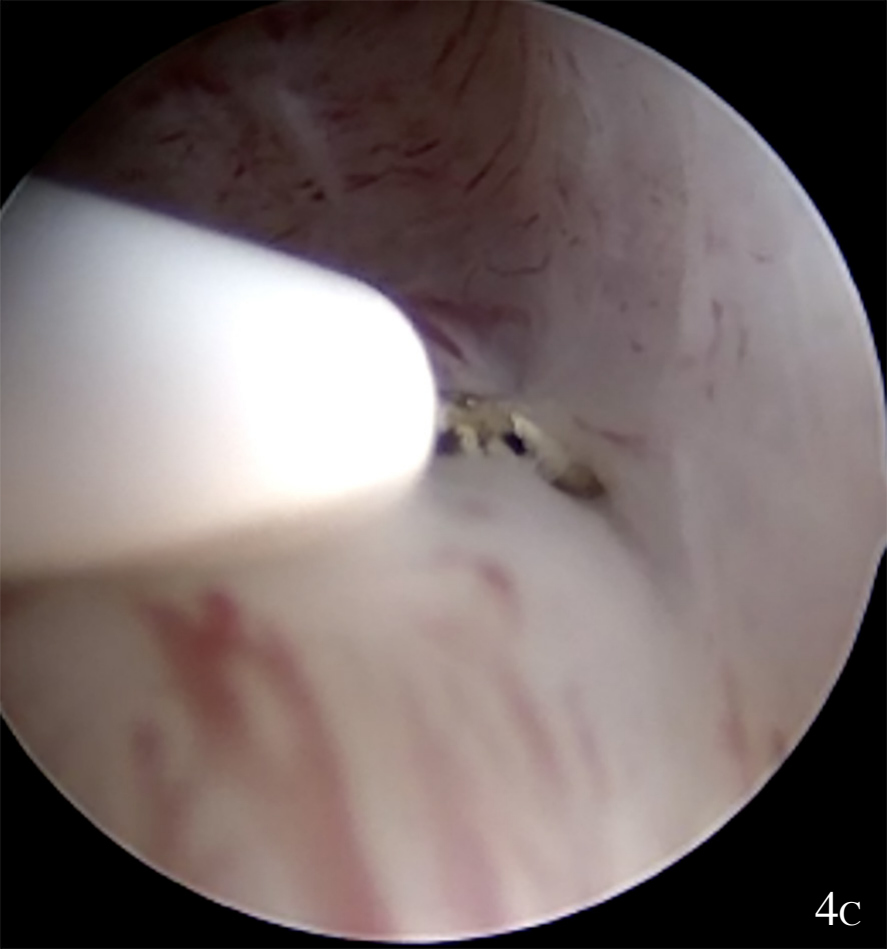
Type 1 fibroid removal using 5Fr electrode. A. Type 1 submucosal fibroid. B. Incision is made to open the fibroid pseudocapsule. C. Connective tissue bands are divided using the electrode.

The activated 5Fr straight electrode is used to wholly, or partially, to circumscribe the endometrium overlying the visible margins of the fibroid, where possible moving the activated electrode in a retrograde direction. Complete circumcision can usually be achieved with more sessile fibroids as the whole distal, endometrial border of the fibroid can be visualised. As described in the preceding sections, once the fibroid capsule has been opened in this way, the myometrial component of the fibroid is free to protrude further into the uterine cavity facilitated by uterine contractions. The inactivated electrode or a 5Fr grasping forceps can be used to mechanically identify the cleavage plane, separating the intramural component of the fibroid from within the underlying myometrium, by breaking the connecting bridges of fascicular myometrial tissue, as described in ‘Cold Loop Myomectomy’ section, thereby delineating the capsule. Precise electrosurgical cuts can then be made under vision where connective tissue attachments of the fibroid to the underlying capsule are seen (Figure 4). Further mechanical manipulation with the inactivated electrode or grasping forceps can be repeated to detach more fibroid, followed by targeted electrosurgical cutting as before. In this way the intramural component of the submucous fibroid becomes increasingly visible as it migrates further into the uterine cavity and becomes more accessible (Istre et al., 2009; Varma et al., 2009; Munro, 2016). These cutting and mechanical steps are systematically repeated until the fibroid is completely enucleated. Removal is as described in section for FIGO type 0 fibroids. Electrosurgical en-bloc removal of FIGO type 1 and 2 fibroids is generally performed in an operating room setting under regional or general anaesthesia due to the longer duration of procedures and the need to manipulate within the myometrium.

#### Fibroids with intramural development (FIGO Type 3)

To date, the surgical technique for treatment of FIGO type 3 fibroids has not been well-defined. A recent study has demonstrated the potential efficacy of operative hysteroscopy as a treatment option for this type of fibroid (Capmas et al., 2016). Hysteroscopic removal of FIGO type 3 fibroids should only be attempted by experienced surgeons because of the increased complexity of the procedure. The technique requires thorough exploratory mapping to elicit their precise location and topographic- anatomical relationships with regard to the uterine cavity. Resectoscopic removal of FIGO type 3 fibroids potentially offers several advantages over conventional laparotomic or laparoscopic approaches. These include less intraoperative and postoperative blood loss, shorter length of stay and rapid recovery with reduced postoperative pain, although the need for a further procedure to complete the resection is more likely.

Resectoscopic slicing of FIGO type 3 fibroids is performed in an operating room setting under regional or general anaesthesia due to the longer duration of procedures and the need to manipulate within the myometrium.

#### Multi-step procedures

All women undergoing hysteroscopic myomectomy should be aware of the chance of incomplete resection and the need for further procedures. This is more likely where there are adverse features, namely maximum diameter above 2cm, large surface area of the uterine cavity covered and >50% myometrial penetration (Hart et al., 1999; Vercellini et al., 1999; Lasmar et al., 2005). The need for multi- step procedures can be minimised with increased operator experience and experience, use of optimal techniques and modalities (see preceding sections) and use of automated fluid management systems (Di Spiezio Sardo et al., 2008; Istre et al., 2009). Pre- operative use of gonadotrophin releasing hormone antagonists (GnRH-a) to inactivate the endometrium and reduce fibroid volume may also increase the feasibility of single step procedures (Istre et al., 2009, Muzii et al., 2010).

It is important that the surgeon and the patient do not consider the inability to complete the procedure as a failure but rather as prudent and safe surgery where visualisation is impaired and cannot be corrected usually because of bleeding and / or a congested endometrium or because the agreed fluid deficit threshold has been reached. The surgeon should be mindful that the subsequent procedures can be easier as some or all of the residual intramural component of the fibroid frequently migrates into the uterine cavity, following cutting into the overlying capsule and concomitant myometrial contractions and uterine shrinkage. Indeed, this observation forms the rationale for pre-operative surgical preparation of submucosal fibroids (“Oppium” technique) (Bettocchi et al., 2009; Haimovich et al., 2013) where FIGO type 1 and 2 fibroids are converted into FIGO type 0 and 1 fibroids, respectively, easing subsequent hysteroscopic resection.

#### Post-operative care

##### Control of post-operative bleeding and pain

The origin of bleeding should be assessed. If arising from cervical trauma then mechanical compression, topical haemostatic agents (e.g. Monsel’s ferric subsulphate solution) or surgical sutures may be used. Intrauterine bleeding is more common following the removal of larger or multiple, FIGO type 1 and 2 fibroids. Emptying the bladder and bimanual compression will normally suffice, but if bleeding persists then uterine tamponade with a balloon catheter (e.g. Foley catheter) for a few hours can be used. If continued bleeding despite these measures, particularly if the procedure was complex, should alert the operator to the possibility of unrecognised uterine trauma requiring further exploratory intervention such as laparoscopy / laparotomy. Similarly, if the patient has excessive post-operative pain that is not controllable with routine opiate and non-opiate analgesics, the risk of uterine perforation and intra-abdominal bleeding or visceral injury should be suspected (Istre et al., 2009).

##### Fluid overload

Fluid deficit thresholds are >1 L for non-isotonic media and >2.5 L for isotonic fluid media (Umranikar et al., 2016; AAGL., 2013). Such deficits may be lower in small women and for those with cardiovascular co-morbidities (Istre et al., 2009). The clinical condition of the patient should be assessed post-procedure if pre-defined fluid deficit thresholds are exceeded, in higher risk patients with co-morbidities and when the procedure is prolonged especially when larger or multiple FIGO type 1 and 2 fibroids have been removed. Oxygen saturation and respiratory rate should be monitored and the patient observed for a productive cough. Where intravasation of excessive fluid (“fluid overload”) is suspected, then specific, evidence-based management protocols produced by the British Society of Gynaecological Endoscopy (BSGE)and the European Society for Gynaecological Endoscopy (ESGE) (Umranikar et al., 2016) and the American Association of Gynaecological Laparoscopists (AAGL) (AAGL., 2013), should be adhered to. The basic approach is to fluid restrict, use diuretics judiciously and observe (including urinary catheterisation to measure output, and measurement of serum urea, electrolytes and O2 saturation) and obtain help from anaesthetic and medical colleagues if required.

##### Adhesion prevention

The de novo formation of intrauterine adhesions (IUAs) can be associated with hysteroscopic myomectomy especially if the fibroid(s) removed covers a relatively large surface area or fibroids on opposing uterine surfaces are removed. Careful, precise and atraumatic surgery is likely to minimise the risk of IUAs. The overall incidence of clinically significant IUAs is thought to be low (Di Spiezio Sardo et al., 2008; Yang et al., 2008). For women requiring future fertility, the formation of intrauterine adhesions (IUAs) may impact adversely on reproductive potential. Thus, preventative strategies should be considered such as the use of hormones to stimulate the endometrium, intrauterine anti-adhesive barriers (e.g. Hyaluronic acid gel) and second look outpatient/office hysteroscopies to detect and lyse newly formed IUAs (Yang et al., 2016). However, in the absence of clear evidence of benefit (Di Spiezio Sardo et al., 2016) on reproductive outcomes, the use of post-operative IUA preventative strategies should be left to the discretion of the operator.

##### Assessing completeness of treatment

Ultrasound examination and second-look hysteroscopy should be considered where complete removal of fibroid tissue is not achieved or is uncertain as a result of suboptimal visualisation, patient tolerance factors (outpatient/office) or curtailment of the procedure due to fluid deficit thresholds being reached. If a small amount of residual fibroid tissue is suspected at most, then an outpatient/office setting is suitable and more convenient to inspect the cavity and remove any residual tissue if feasible or schedule an operating room repeat procedure. Ideally, second-look procedures should be undertaken within 6- 8 weeks of the index procedure.

## Conclusion

These ESGE Good Practice recommendations provide guidance to help optimise the care of women undergoing surgical removal of submucosal uterine fibroids using a hysteroscopic approach. Optimal clinical outcomes necessitate an understanding of the indications for hysteroscopic myomectomy, diagnostic workup and treatment planning as well as the importance of good surgical technique, appropriate use of available technologies and fluid management.

## Recommendations

- Women should be counselled about medical and hysteroscopic surgical management and submucosal fibroids, including their relative risks and benefits.
- Pre-operative assessment using imaging and/ or hysteroscopy is of key importance to characterise the submucosal fibroid(s) to help plan hysteroscopic myomectomy, including patient counselling, the likelihood of multi- stage procedures, the need for medical/surgical preparation, selection of viable technologies and appropriate treatment setting(s).
- Understanding fluid management is essential to optimise the safety and feasibility of hysteroscopic myomectomy. Automated fluid management systems should be used to maintain intrauterine pressures and visualisation and monitor fluid deficit.
- Identification and delineation of the plane between the fibroid and myometrium (pseudocapsule) are important to ensure complete enucleation (removal) of the fibroid.
- The size and intramural penetration of the submucosal fibroid(s) are the main factors to consider when deciding about the best surgical approach: technologies (mechanical, electrosurgical and mHTR), techniques (slicing, en-bloc removal, cold loop, vaporisation, mHTR) and setting.
- Where possible, smaller diameter operative hysteroscopes should be used to minimize the risk of cervical trauma and increase the feasibility of the office/outpatient setting.
- Consider post-operative imaging / outpatient hysteroscopy to assess the completeness of treatment and intrauterine adhesions.

## What does this mean for patients?

These recommendations have been produced by the European Society for Gynaecological Endoscopy (ESGE) Uterine Fibroids Working Group. The objective of the Working Group was to develop guidance based on the best available evidence and expert opinion for the surgical treatment of uterine fibroids. The recommendations are intended to provide information for clinicians involved in the care of women where surgical treatment of fibroids is indicated because of their interference with fertility and pregnancy or because of ongoing pressure, pain or bleeding symptoms.

The working group has produced two documents describing procedures to perform myomectomy utilising both abdominal (part 1) and hysteroscopic (part 2) routes. This second document (part 2) focuses on hysteroscopic myomectomy and considers preoperative work-up, and intraoperative techniques including fluid management, the available technologies and treatment settings. Postoperative management and follow up is also covered.
